# Wind power variation by wind veer characteristics with two wind farms

**DOI:** 10.1038/s41598-023-37957-6

**Published:** 2023-07-04

**Authors:** Undarmaa Tumenbayar, Kyungnam Ko

**Affiliations:** 1 Multidisciplinary Graduate School Program for Wind Energy, Jeju National University, 102 Jejudaehakro, Jeju, 63243 South Korea; 2 Department of Electrical and Energy Engineering, Jeju National University, 102 Jejudaehakro, Jeju, 63243 South Korea

**Keywords:** Renewable energy, Wind energy

## Abstract

To clarify the wind veer characteristics with height and their effect on the wind turbine power outputs, an investigation was carried out at the wind farms with complex and simple terrains. A 2 MW and a 1.5 MW wind turbine were tested, each having an 80 m tall met mast and a ground lidar to capture wind veering. Wind veer conditions were divided into four types based on wind direction changes with height. The power deviation coefficient (PDC) and the revenue differences for the four types were derived from the estimated electric productions. As a result, the wind veer angle across turbine rotors were more significant at the complex site than at the simple site. For the two sites, the PDC values ranged from − 3.90 to 4.21% depending on the four types, which led to a 20-year revenue variation of − 274,750–423,670 USD/MW.

## Introduction

The power outputs of a wind turbine have been known to be affected by the factors of incoming wind speed, wind direction, atmospheric stability, turbulence, air density, wind shear, wind veer and so on. The factors influencing wind turbine power production have a diurnal cycle due to the surface heat from the sun^1–3^. Every site has unique conditions, thus wind measurement campaigns should be carried out to estimate accurate electric power productions of wind turbines at a given site. Over the last decades, the investigations on the effect of atmospheric stability conditions on wind power productions have been conducted by many researchers^4–6^. Recently, the wind directional change with height has been given much attention by wind energy industries since it influences turbine productions, however, this relation is not widely studied compared to other atmospheric parameters.

When the wind direction changes with height above the ground, it is referred to as wind directional shear or wind veer. It is a veering wind when the wind direction changes clockwise with height and it is a backing wind when the wind direction changes counter clockwise with height^7, 8^. The veering wind generally occurs in the stable atmospheric condition, while the backing wind occurs in the unstable atmospheric condition^9, 10^. Terrain characteristics also have an influence on directional wind shear. The wind veer angles of the hilly terrain condition were much higher than those of the open sea condition^11^. Also, the wind veer angles on the lower part of the rotor tend to have higher values than those on the upper part on sites with complex terrain^12^.

A large wind turbine could experience a comparatively large wind directional difference across the rotor. Several studies have been carried out to determine the influence of the directional shear on wind turbine power performance. It was reported that wind directional differences had an effect on wind turbine power output^13, 14^ as well as mechanical load^15, 16^. The effect of the overall wind veer not divided by veering and backing winds has been analyzed by Saint-Drenan et al.^17^ and Bardal et al.^18^, whose results showed that the effect of wind veer on turbine power performance was comparably small. The impact of power production has been studied with the feature of veering and backing winds^19, 20^. It was pointed out that a small clockwise directional shear caused an increase of the power output while a counter clockwise directional shear resulted in underperformance. Murphy et al.^21^ also obtained that a veering wind led to power gain while a backing wind resulted in power loss.

Although most of the studies on wind veer characteristics were carried out in terms of veering and backing winds, various wind veer conditions across the rotor should be considered since topographical and roughness conditions will vary at many sites. Recently, the power deviation coefficient was computed in accordance with the four types with veering conditions at upper and lower areas from the turbine hub height^22^. It was found that the power deviation coefficient was in a range from − 6.5 to 1.6% for the four types. Further investigation is necessary on wind veer characteristics with various wind farm sites.

This work aims to investigate the relationship between wind veer characteristics and the power output at two wind farm sites: one near a large landfill and the other located at the seaside. The wind veer was classified by four types depending on the wind direction change across the rotor with height above the ground. The power curves from the four types were compared with that under no veer condition to reveal the power deviation. The revenue differences along with various electricity productions with the four types were analyzed according to annual average wind speeds from 4 to 11 m/s.

## Test setup and data filtering

### Test site

**Figure 1:**
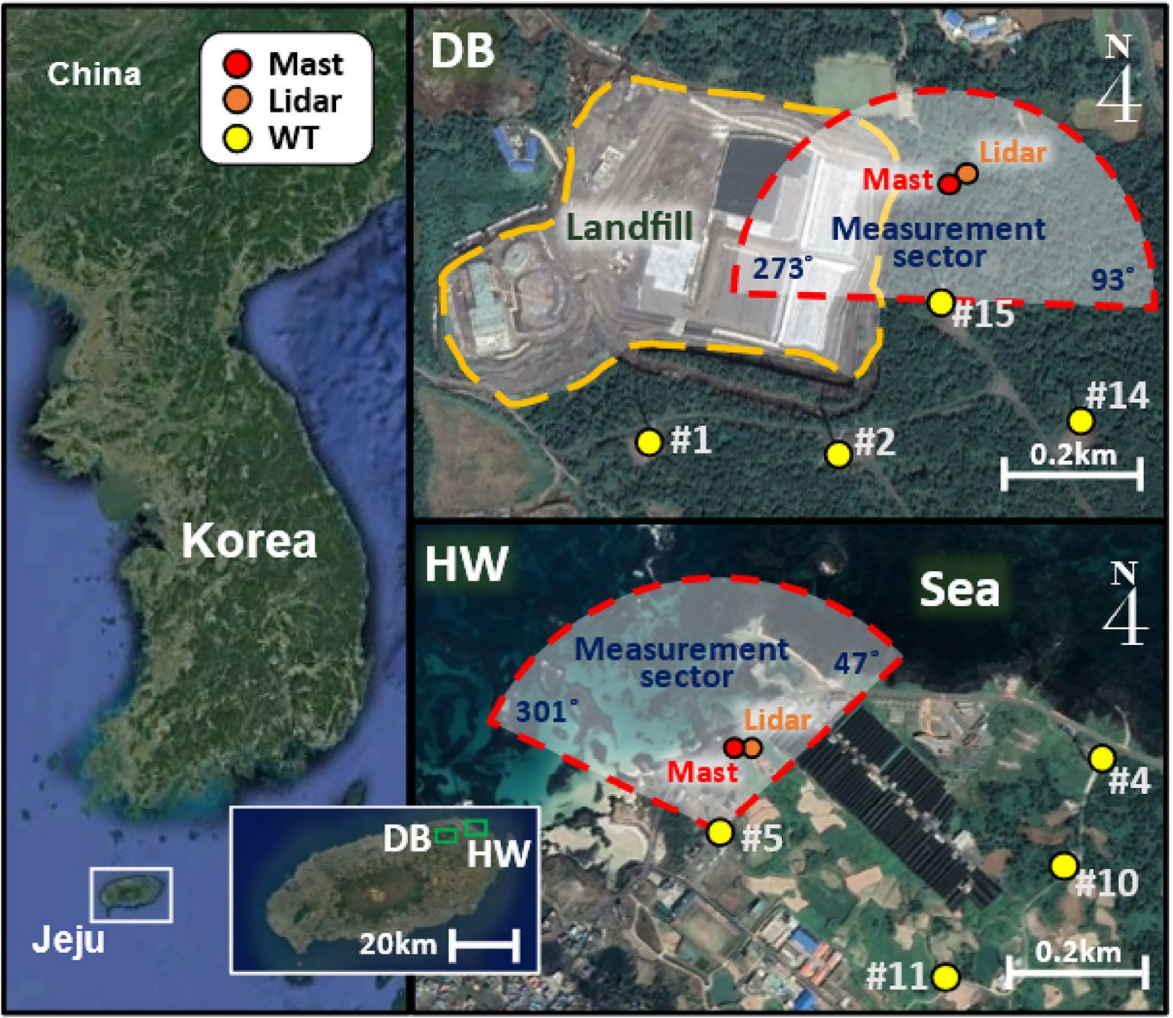
Location of Jeju Island and layout of the test wind farms as well as measurement instruments. (Geographic map was edited with Microsoft Office powerpoint 2016, https://www.microsoft.com/ko-kr/microsoft-365/powerpoint and satellite images by Google Earth Version 9, http://earth.google.com/).

This work was carried out at wind farms on Jeju Island which is located off the southern part of the Korean peninsula. Figure 1 shows the location of the Dongbok (DB) and the Haengwon (HW) wind farms as well as the layout of the measurement instruments and neighboring wind turbines. Both the DB and the HW sites are located about 10 km apart in the north-eastern part of Jeju Island. A wind turbine, a met mast and a ground lidar were used for measuring the wind conditions. There is a landfill located near the test wind turbine at the DB site while the test wind turbine at the HW site was situated at the seaside. In order to consider the wake from neighboring wind turbines, the measurement sector of each site was derived by the following equation^23^:

1 $$\theta =1.3\mathrm{Arctan}\left(2.5D/L+0.15\right)+10$$

where D is the rotor diameter and L is the distance between the test wind turbine and the neighboring turbines.

The measurement sectors were from 273° to 93° at the DB site and 301° to 47° at the HW site, respectively. The comparatively complex terrain with the ruggedness index (RIX)^24^ value of 0.87% was found in the measurement sector due to the nearby landfill at the DB site. On the other hand, the measurement sector of the HW site had an RIX value of 0.00% due to the very simple terrain and the seaside.

### Test instruments

Table 1 lists the specification of the measurement instruments and the test wind turbines. The 80 m tall met masts were installed 220 m and 212 m away from each test wind turbine at the DB and the HW site, respectively. Each ground lidar was situated close to each met mast. The wind conditions at the hub height were measured by a Thies first class anemometer and wind vane which were mounted on the met masts. The ground lidar measured wind conditions across the rotor at both sites. The 10-min average wind measurements were analyzed with power production data from the test wind turbines. The wind conditions were measured for about 6 months from the 2nd of October, 2018, to the 19th of April, 2019, at the DB site, and for about 9 months from the 17th of June, 2016, to the 2nd of March, 2017, at the HW site.

**Table 1:** Specification of the measurement instruments and test wind turbines.

Category	DB site	HW site
Met mast		
Cup anemometer	Thies first class advanced	
Wind vane	Thies first class advanced	
Thermometer	PT 100 CLA	
Hygrometer	Potronic hygrometer	
Barometer	P-GE 6/11	
Ground lidar		
Model	Windcube V2	
Measurement range	40–200 m	
Sampling rate	1 Hz	
No. of measurement height	12	
Analyzed measurement points	40, 80, 124 m	40, 70, 105 m
FCR module	On	Off
Wind turbine		
Model	HJWT 2000	HJWT 1500
Rated power	2000 kw	1500 kw
Hub height	80 m	70 m
Rotor diameter	87 m	70 m
Cut-in/rated/cut-out wind speed	3/12/25 m/s	4/13/25 m/s
SCADA system	Gateway	

The air temperature, the humidity and the atmospheric pressure data were used for normalization of the specific air density at the two sites. The data were measured at a height of 75 m at both sites using the meteorological sensors listed in Table 1. The ground lidar used was the Windcube v2 developed by Leosphere. It has five laser beams that are emitted vertically to measure wind conditions at 12 points from 40 to 200 m above the ground. The 2 MW and 1.5 MW wind turbines with different hub heights and rotor diameters were tested at the DB and the HW sites, respectively. The flow complexity recognition (FCR)^25^ module for ground lidar was used at the DB site to correct the wind measurement error caused by the terrain complexity. However, it was not used at the HW site as the measurement site was considered to have a simple terrain. The power outputs from the test wind turbines were collected through the Gateway Supervisory Control and Data Acquisition (SCADA) system.

### Data fileting

The wind data with carrier-to-noise ratios (CNR) of less than − 23 db or with an availability of less than 80% or data measured by lidar in abnormal operation were rejected according to the study by Kim et al.^26^ and the ground lidar user manual^27^. Also, when the wind turbines produce electric power significantly lower or higher than the expected power, or when the wind turbines were in abnormal operation, or when the turbine yaw misalignment was over 30°^28^, the wind measurements and the power data were removed from the database. Additionally, not only the wind speeds lower than the cut-in and higher than the cut-out speeds but also the data outside the measurement sector were discarded for the analysis.

After the filtering process, the remaining data were approximately 50.7% for the DB site and 26.7% for the HW site, respectively, which was named as all data for formal analysis in this work.

## Results and discussions

### Atmospheric condition and lidar accuracy check

The fundamental analysis on wind conditions was performed for the two sites. The atmospheric stability conditions were evaluated in terms of the bulk Richardson number (Ri_b_) by the following equation^29, 30^:

2 $${Ri}_{b}=\frac{gz({T}_{1}-{T}_{2})}{(273.15+ {T}_{1}){V}^{2}}$$

where V is the wind speed measured at the height z, and g is the acceleration due to gravity. T_1_ and T_2_ are the air temperatures at the 75 m and 3 m heights at both sites in this work, respectively. The atmosphere is under an unstable condition when the value of Ri_b_ is lower than − 0.02. When the value is higher than 0.02, the atmosphere is under a stable condition. Also, the values between − 0.02 and 0.02 correspond to a neutral atmospheric condition. The atmospheric stability conditions at the DB site were categorized into comparatively fair ratios with 34.9%, 41.5% and 23.5% for stable, neutral and unstable conditions, respectively. On the other hand, the HW site had prevalent neutral conditions with 68.1%, followed by stable and unstable conditions with 20.5% and 11.4%, respectively. The two sites had typical features of the onshore and offshore atmospheric stability conditions, which is a similar portion for the land and prevailing condition of neutral for the sea^31, 32^.

**Figure 2:**
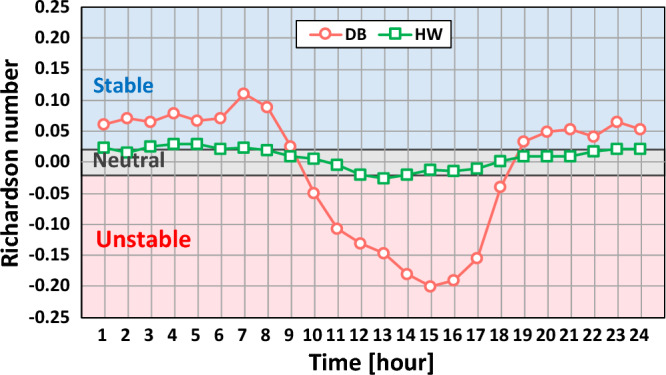
Diurnal variation of atmospheric stability.

Figure 2 represents the diurnal variation of the atmospheric stability condition at the two sites. The values of Ri_b_ at the DB site had higher fluctuation between the daytime and nighttime. On the other hand, those at the HW site were mostly observed in and around the neutral conditions with very small fluctuations throughout the day. The stable atmospheric condition mostly appeared during the nighttime while the unstable atmospheric condition occurred mostly during the daytime at the two sites. This trend was the same as the studies of Radünz et al.^33^ and Ryu et al.^34^.

**Figure 3:**
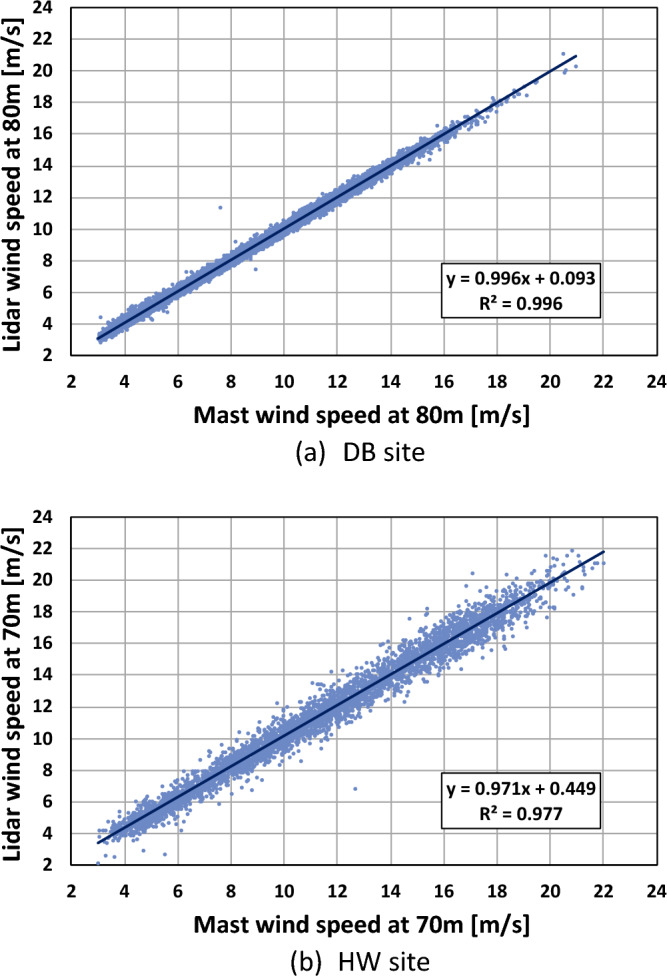
Linear regression analysis between the met mast and ground lidar wind speeds.

The results of the linear regression analysis between the met mast and the ground lidar wind speeds at the two sites are displayed in Fig. 3. The data points were more scattered at the HW site than at the DB site, because the FCR module was used at the DB site with complex terrain, while the HW site with simple terrain did not need the module. The wind speed correlation was very high with the linear line slopes of 0.996 and 0.971 and the coefficients of determination (R^2^) of 0.996 and 0.977 at the DB and the HW sites, respectively. Although more scattering was observed at the HW site, the difference between the values of R^2^ for the two sites were very low. This meant that very small measurement error occurred at the HW site so that the measurements at both sites were used without any data correction in this work.

**Figure 4:**
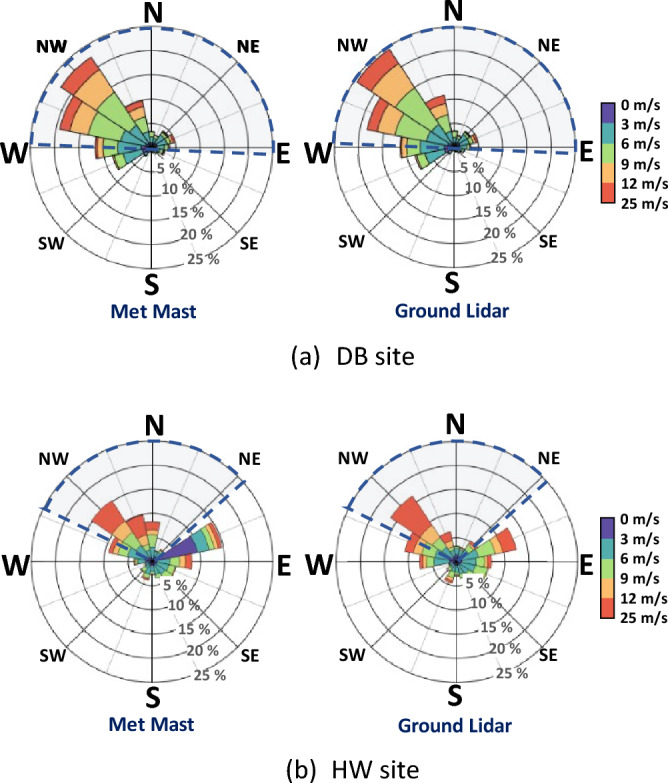
Wind roses of met mast and ground lidar wind directions. The navy dotted lines highlight the measurement sector.

Figure 4 shows the wind roses at the hub heights from the met mast and the ground lidar at the two sites. The wind roses from the two instruments at the DB site showed the same northwest prevailing wind direction with overall similar directional distribution because of the use of the FCR module. On the other hand, the wind roses at the HW site had a slightly different directional distribution trend, while having the same prevailing wind direction of the northwest and east-northeast. The small difference of wind direction distribution at the HW site was caused by the absence of the FCR module. However, the small difference is considered to be negligible for the purpose of this work.

### Wind veer analysis

According to the characteristics of the wind direction change with height above the ground, the wind veering phenomenon can be categorized as a veering or backing wind. When the wind direction shifts clockwise with an increase of height, the wind is referred to as a veering wind. On the other hand, it is a backing wind when the wind direction changes counter clockwise with an increase of height^35^. The wind veer is evaluated by the following equation^36^:

3 $$Wind\,veer=\frac{\Delta\uptheta }{\Delta Z} \,(\mathrm{deg}/\mathrm{m})$$

where ΔZ is the measurement height difference and Δθ is the wind directional difference from the upper and the lower measurement heights, respectively.

The wind veer was analyzed using the wind directions at the measurement heights of 40 m, 80 m and 124 m at the DB site and 40 m, 70 m and 115 m at the HW site, respectively. Table 2 presents the frequency of the overall wind veer at the two sites. The overall wind veer was calculated using the highest and the lowest measurement heights which was 124 m and 40 m at the DB site and 115 m and 40 m at the HW site, respectively. The wind veer distribution of the DB site were mostly concentrated on the range of − 0.07 to 0.23 deg/m, while 75.8% of all data were gathered in the range of − 0.03 to 0.03 deg/m at the HW site. The DB site had the veering wind of 83.1% and the backing wind of 16.9%, while the veering and the backing winds were found to be 53.1% and 46.9% at the HW site, respectively. The mean wind veer using all data were 0.052 deg/m and 0.004 deg/m for the DB and the HW sites, respectively. The distribution of the wind veer angle at both sites followed a normal distribution, which was in good agreement with studies of Wharton et al.^12^ and Rybchuk et al.^37^.

**Table 2:** Overall wind veer distribution.

Wind veer value (deg/m)			DB site (%)	HW site (%)
Median	Min	Max		
− 0.30	− 0.32	− 0.27	0.13	0.0
− 0.25	− 0.27	− 0.22	0.14	0.2
− 0.20	− 0.22	− 0.17	0.16	0.3
− 0.15	− 0.17	− 0.12	0.33	0.5
− 0.10	− 0.12	− 0.07	0.78	1.2
− 0.05	− 0.07	− 0.02	4.66	8.4
0.00	− 0.03	0.03	27.64	75.8
0.05	0.03	0.08	38.67	9.0
0.10	0.08	0.13	15.63	2.1
0.15	0.13	0.18	3.74	0.9
0.20	0.18	0.23	1.05	0.6
0.25	0.23	0.28	0.50	0.5
0.30	0.28	0.33	0.31	0.2

**Figure 5:**
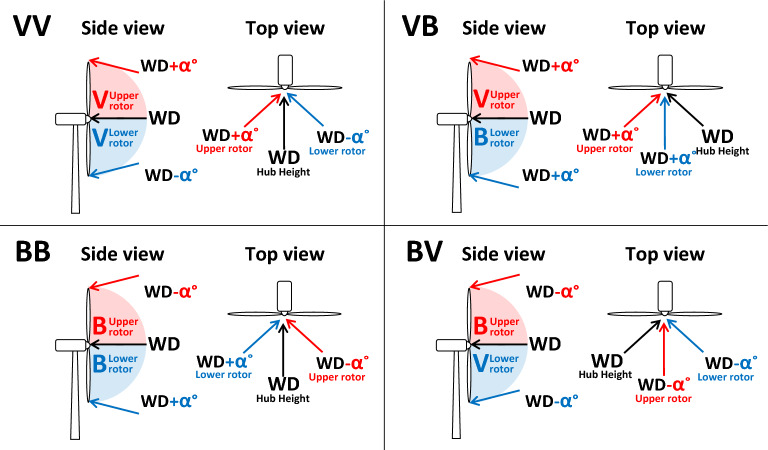
Schematic illustration of wind veer types. α is wind direction shifting angle with height. (Schematic figure was created with Microsoft Office powerpoint 2016, https://www.microsoft.com/ko-kr/microsoft-365/powerpoint).

The wind veer types were divided by the wind direction changes from the upper part of the swept area to the hub height and from the hub height to the lower part of the swept area^22^. The four types of wind veer conditions are illustrated in Fig. 5. The α in the figure represents the wind direction shifting angle with height. The four types are VV, veering from the upper part to the hub height and also from the hub height to the lower part, VB, veering from the upper part to the hub height and backing from the hub height to the lower part, BB, backing from the upper part to the hub height and also from the hub height to the lower part and BV, backing from the upper part to the hub height and veering from the hub height to the lower part.

**Figure 6:**
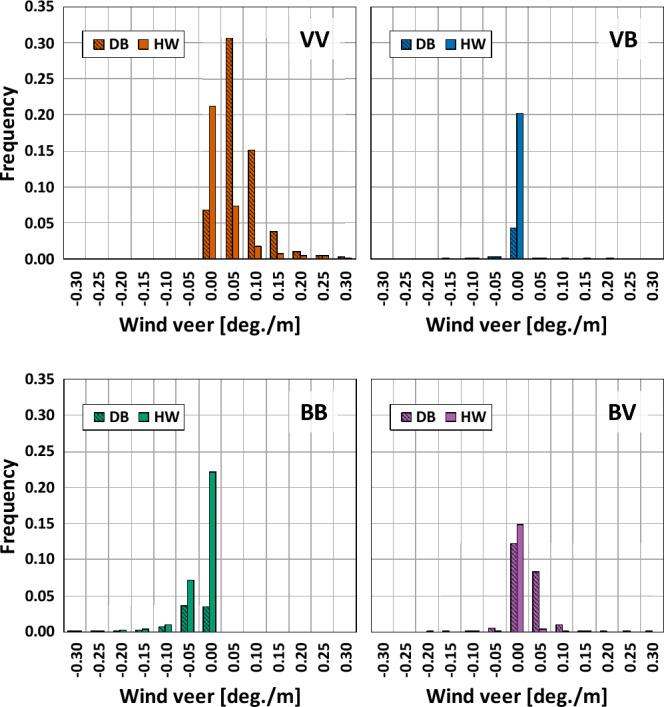
Wind veer distribution of the four types.

Figure 6 shows wind veer distribution of the four types at both sites. The types VV and BB had only positive and negative values with average values of 0.092 deg/m and − 0.066 deg/m at the DB site, and 0.035 deg/m and − 0.026 deg/m at the HW site, respectively. Both positive and negative values were found in the cases of the types VB and BV. The types VB and BV were in the range of − 0.17 to 0.05 deg/m and − 0.13 to 0.33 deg/m at the DB site, respectively, while those ranged from − 0.11 to 0.19 deg/m and from − 0.20 to 0.16 deg/m at the HW site, respectively. Overall, the wind veer distribution of the DB site was wider than that of the HW site, and higher average values of wind veer angles were found at the DB site compared to the HW site.

**Figure 7:**
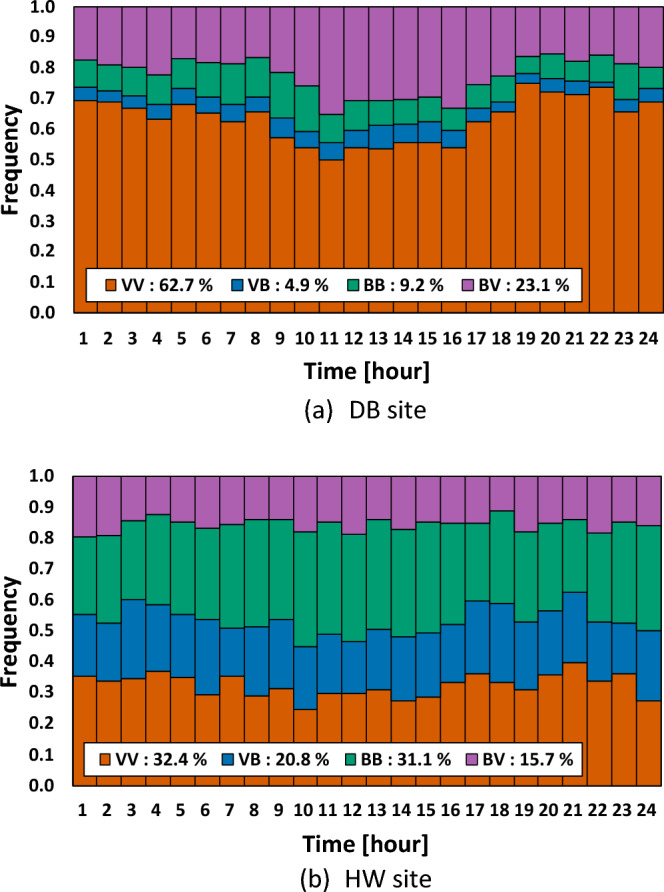
Total and diurnal frequencies of the four types.

Figure 7 shows total and diurnal frequencies of the four types. At the DB site, the total frequency of type VV was the highest with 62.7% followed by type BV with 23.1%, while the frequency of type VB was the lowest with 4.9%. Type BB was less than 10%. On the other hand, at the HW site, there was no total frequency excessing 50% among the four types. The proportions of the types VV and BB were nearly the same frequency with 32.4% and 31.1%, respectively. Type VB took third place with 20.8% followed by type BV with 15.7%.

As for the diurnal frequency, at the DB site, the types VV and BV exhibited opposite diurnal variations, while the other two types had similar diurnal frequencies throughout the day. In contrast, at the HW site, there were small variations observed for all the types for diurnal frequency regardless of daytime or nighttime. This result was considered to be caused by the considerable diurnal variation of atmospheric stability at the DB site and little variation at the HW site, as shown in Fig. 2.

**Figure 8:**
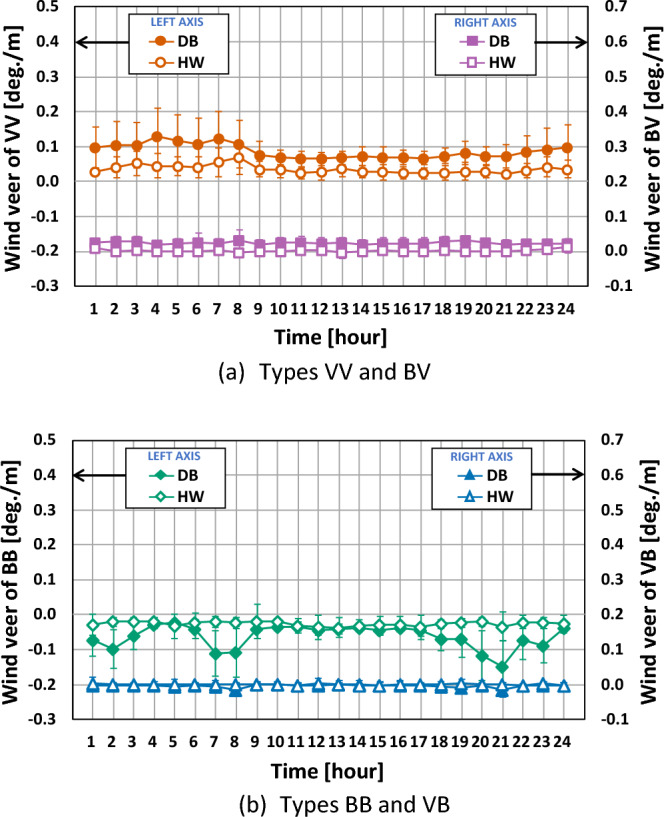
Diurnal variation of wind veer according to the four types. The error bar corresponds to one standard deviation.

Figure 8 represents the diurnal variation of the wind veer for the four types at both sites. The wind veer values of types VV, BV and BB at the DB site were higher than those at the HW site. The wind veer values of type VB at both sites were close to zero and had very low fluctuation throughout the day. This could have been caused by the relatively small frequency of 4.9% at the DB site, which could not be sufficient to recognize the difference of the wind veer angle.

The wind veer values and fluctuations of the types VV and BB at the DB site were higher during the nighttime than during the daytime. On the other hand, little change of wind veer values and fluctuation were found for the remaining two types, VB and BV, throughout the day at the HW site, since the two types contained both positive and negative wind veer angles, as shown in Fig. 6, causing neutralization.

**Figure 9:**
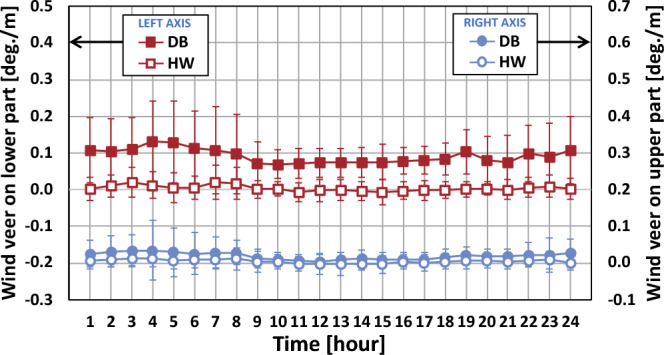
Diurnal variation of wind veer on upper and lower parts of the rotor. The error bar corresponds to one standard deviation.

The diurnal variation of wind veer on the upper and lower parts of the rotor are compared in Fig. 9. The wind veer values on the upper part were calculated using the measured wind directions at the heights of 124 m and 80 m at the DB site and 115 m and 70 m at the HW site, respectively, while those on the lower part were derived using the wind direction at 80 m and 40 m at the DB site and 70 m and 40 m at the HW site, respectively. The lower part had higher wind veer values as well as fluctuations compared to the upper part at the both sites. The wind veer value on the upper part was close to zero throughout the day at the two sites. The diurnal trend of wind veer at the two sites were similar to the result by Gao et al.^22^.

The comparatively complex terrain at the DB site had more effect on wind veer on the upper and lower parts than the very simple terrain and the seaside at the HW site. The wind veer values on the lower part at the DB site were more influenced by the topographical condition compared to those on the upper part. From the error bar at both sites, the wind veer fluctuation of the lower part was more significant than the upper part. Therefore, the wind veer shifting angle with height could rely on the topographical condition. In other words, a complex terrain could have more significant wind veer angle than a simple terrain and furthermore the shifting angle could be bigger at the lower part than at the upper part of a turbine rotor. This finding was similar to the work of Wharton et al.^12^.

**Figure 10:**
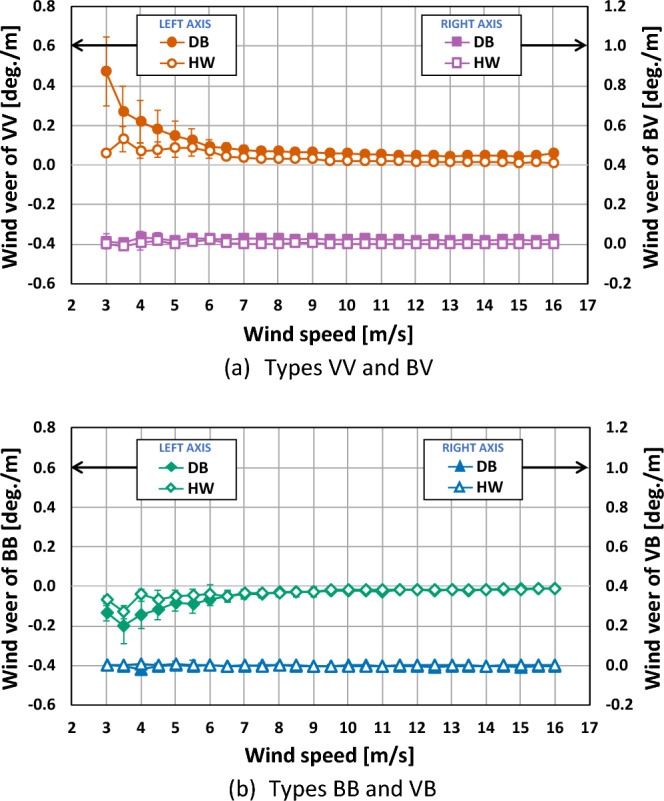
Wind veer variation with wind speeds for the four types. The error bar corresponds to one standard deviation.

The wind veer variation with wind speeds for the four cases are presented in Fig. 10. In case of the types VV and BB, as wind speeds increased, the wind veer values and the fluctuation decreased. This could be because high wind speeds usually mechanically more mix momentum compared to lower wind speeds in wind flow^21^. For this reason, the types VV and BB also had higher values and fluctuations at wind speeds less than 6 m/s. On the other hand, the types VB and BV showed small wind veer values as well as little fluctuations at all wind speeds due to the neutralization effect.

**Figure 11:**
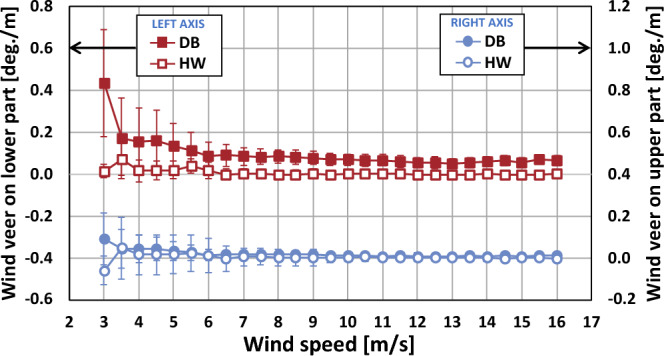
Wind veer variation according to upper and lower parts of the rotor. The error bar corresponds to one standard deviation.

The wind veer variation with wind speeds for the upper and the lower part of the rotor are represented in Fig. 11. The wind veer along with wind speeds on the lower part had higher values at the DB site than at the HW site. That is, the DB site with complex terrain had a much higher effect on the wind veer on the lower part than the HW site with simple terrain regardless of wind speeds. The wind veers according to wind speeds on the upper part at both sites were similar to each other with values close to zero, which meant that even the complex terrain of the DB site did little to influence the wind veering condition on the upper part, similar to the simple terrain of the HW site. Overall, the wind veer values and the fluctuations were reduced with an increase of wind speed, which was in good agreement with the works of Murphy et al.^21^ and Shu et al.^11^.

### Power output

The measured wind speeds were normalized to standard air density and the power curves of the test wind turbines were drawn in compliance with IEC 61400-12-1 3rd edition^38^. The power curve under no wind veer condition was derived using the data points that had wind veer values within ± 0.01 deg/m^22^. The wind data points with no veer condition accounted for 11.3% and 42.5% of all data points at the DB and the HW sites, respectively. The power curve under no veer condition was used as a reference for further analysis in this work.

**Figure 12:**
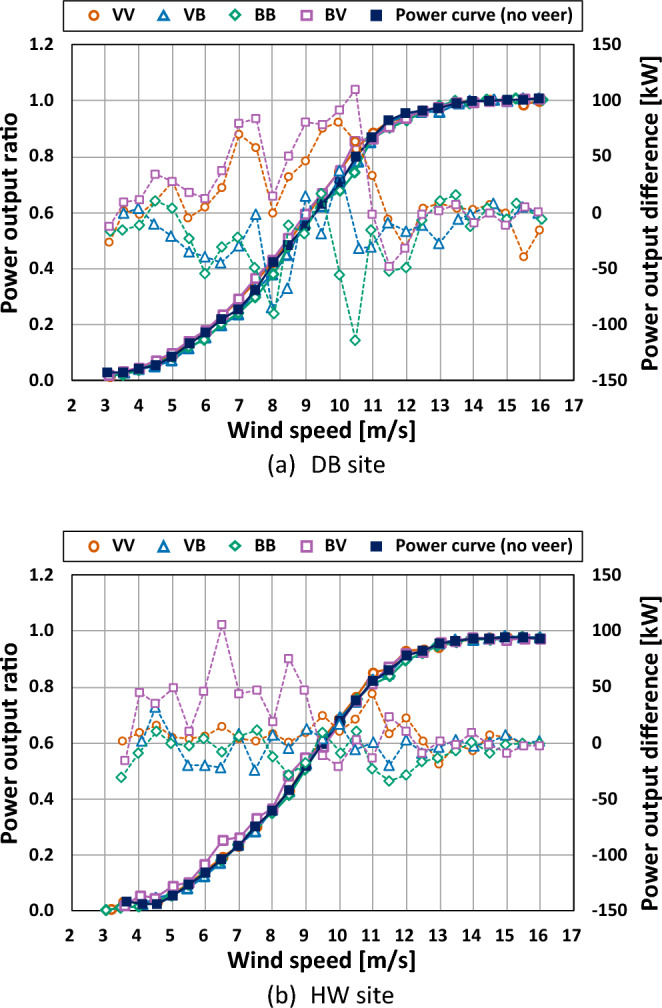
Power curve and power output difference according to the four types.

Figure 12 shows the power output ratio and the difference with wind speeds for the four types. The power output ratio comes from the power output by the rated power output. The power output difference means the power output of each type minus that under no veer condition. The fluctuation of power outputs became significant with an increase of the slope of the power curve. The power outputs of the types VV and BV were mostly higher than those under no veer condition while types VB and BB had roughly lower power outputs than those under no veer condition at the two sites. Higher fluctuation of power output difference was observed at the DB site than at the HW site. In other words, atmospheric stability and topographical conditions had an influence on the power curves as well as the wind veer characteristics.

The power deviation coefficient (PDC) was calculated by the following equation^22^:

4 $$PDC=\frac{\sum {P}_{each\, type}-\sum {P}_{no \,veer}}{\sum {P}_{no \,veer}} \times 100{\% }\,({\%})$$

where P_each type_ is power outputs of each type and P_no veer_ is those under no veer condition.

The PDC was computed for power outputs before the rated wind speed to minimize the neutralization by almost the same power outputs after the rated wind speed. The value of the PDC indicates power gain or power loss depending on the outputs higher or lower than the zero.

**Figure 13:**
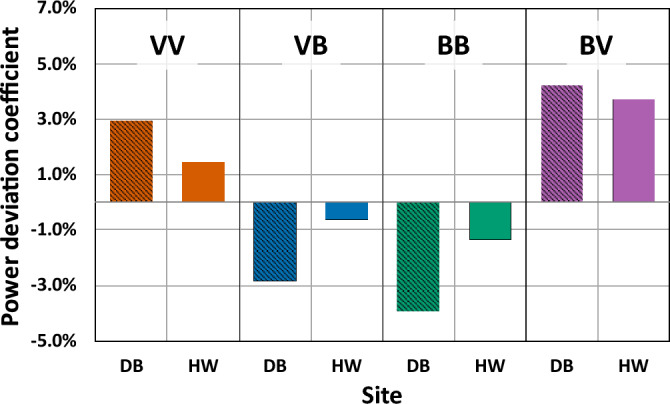
PDC for four types.

Figure 13 shows the PDC values of the four types at the two sites. The types VV and BV had positive values of 2.97% and 4.21% at the DB site and 1.45% and 3.70% at the HW site, respectively, which were a power gain. On the other hand, types VB and BB resulted in power loss with negative values of − 2.85% and − 3.90% at the DB site and − 0.62% and − 1.34% at the HW site, respectively. The result of the PDC for types VV and BB was in good agreement with the studies by Wagner et al.^19^ and Murphy et al.^21^. On the other hand, Gao et al.^22^ and Gomez et al.^20^ reported that the veering wind led to power loss while power gain was found with the backing wind. Accordingly, the finding about power gain and loss in this work was very site specific and further research is needed.

### Economic analysis

The revenue difference by electric productions from the four types was evaluated at various annual average wind speeds. The revenue difference was calculated by the following equation:

5 $$Revenue \,difference=\frac{\left({AEP}_{each\, type}-{AEP}_{no \,veer}\right)\times \left(REC+SMP\right)\times Y}{{P}_{rated}}\, (\mathrm{USD}/\mathrm{MW})$$

where AEP_each type_ and AEP_no veer_ are annual energy production (AEP) of each type and that under no veer condition, respectively. REC is the renewable energy certificate price, SMP is the system marginal price, and Y is operating years. Prated is the rated power of the test wind turbines for normalization.

The AEP was calculated using the combination of a Rayleigh distribution for annual average wind speeds from 4 to 11 m/s and measured power curves according to the IEC 61400-12-1 3rd edition^38^. The fixed price for summation of REC and SMP was 135.9 US dollars (USD) per MWh as of January 2023 in South Korea, which was applied to this work.

**Figure 14:**
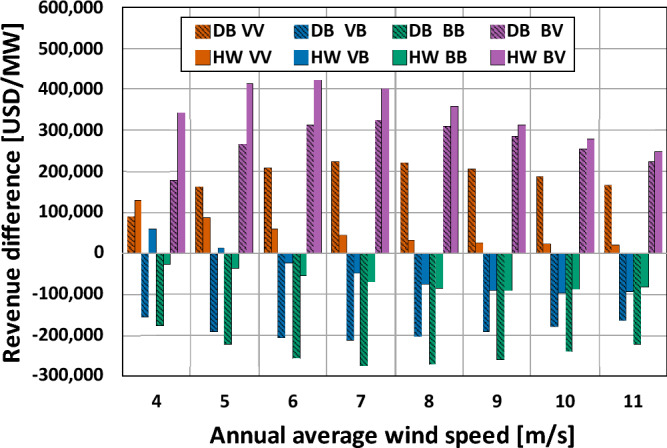
20-year revenue difference with annual average wind speed.

Figure 14 shows the 20-year revenue difference with annual average wind speed for the four types. The revenue differences of types VV and BV at both sites were all positive meaning revenue gain, while those of types VB and BB were mostly negative corresponding to revenue loss. During the 20-year operating period, the revenue differences per MW were varied from − 274,750 to 423,670 USD depending on the annual average wind speeds at the two sites. Among all annual average wind speeds, the revenue differences were the highest at the wind speeds of 6 m/s and 7 m/s, while those were the lowest at the wind speeds of 4 m/s and 11 m/s.

The highest revenue difference was found at type BV at the HW site, which resulted from the corresponding higher power output difference, as shown in Fig. 12. As for the remaining revenue differences, the variation was more significant at the DB site than at the HW site, except the wind speed of 4 m/s.

## Conclusions

The results of this study can be summarized as follows:

- An 83.1% of dominant veering wind was observed at the DB site. The HW site had similar proportions of veering and backing winds with 53.1% and 46.9%, respectively. The type VV had the highest frequency of 62.7%, while the type VB was the smallest with 4.9% at the DB site. The frequencies of four types, VV, VB, BB and BV were 32.4%, 20.8%, 31.1% and 15.7% at the HW site, respectively.
- The topographical condition had more influence on wind veer characteristics at the lower part than the upper part at the DB site with comparatively complex terrain, while those effects were small at the HW site with simple terrain and the seaside.
- In this work, for both sites, the types VV and BV had positive PDC values ranging from 1.45 to 4.21% resulting in a power gain. On the other hand, the types VB and BB resulted in negative PDC values ranging from − 0.62 to − 3.90%, which led to power loss. More variation in PDC caused by more significant wind veering was found at the DB site with comparatively complex terrain than at the HW site with simple terrain.
- The highest revenue differences at both sites occurred at 6 m/s and 7 m/s, in which the types VV and BV had a positive revenue difference resulting from positive PDC, while the types BB and VB had a negative revenue difference coming from negative PDC. The 20-year revenue differences at the two sites were in the range of − 274,750 to 423,670 USD/MW relying on the four types of wind veer at the annual average wind speeds of 4 to 11 m/s.

## Data Availability

Data are available from the corresponding author upon reasonable request and with the permission of Jeju Energy Corporation.
